# Thrombotic thrombocytopenic purpura with neurological impairment: A Review

**DOI:** 10.1097/MD.0000000000031851

**Published:** 2022-12-09

**Authors:** Hui Zhu, Jing-Yao Liu

**Affiliations:** a Department of Neurology, The First Hospital, Jilin University, Changchun, China.

**Keywords:** autoimmune diseases, neurologic complications, spinal cord injury, thrombotic thrombocytopenic purpura (TTP)

## Abstract

The last 2 decades have witnessed considerable advances in our understanding of thrombotic thrombocytopenic purpura (TTP). However, there is still some ambiguity regarding the precise nature of this disease, especially with respect to nervous system involvement and the correct nomenclature. This article seeks to summarize the clinical manifestations of TTP and the associated diseases. We describe TTP complicated with cerebrovascular disease, spinal cord injury, posterior reversible encephalopathy syndrome (PRES), anxious-depressive symptoms, and cognitive decline. TTP with spinal cord injury is rarely reported. For better clarity, we discuss the case of a 57-year-old woman who was diagnosed with neuromyelitis optica spectrum disease (NMOSD) with atypical TTP. The concurrent occurrence of NMOSD and TTP in this patient is consistent with the characteristics of acquired autoimmunity. We highlight the importance of early recognition of TTP in patients with atypical presentation who may not have the expected clinical or laboratory findings. This is particularly important in TTP patients with other concomitant autoimmune diseases or age-related comorbid conditions.

## 1. Introduction

Thrombotic thrombocytopenic purpura (TTP) is caused by severe deficiency of a thrombospondin type 1 motif, member 13 (ADAMTS13).^[1]^ These patients commonly exhibit neurological involvement,^[2]^ including stroke, epilepsy, spinal cord injury, or posterior reversible encephalopathy syndrome (PRES). Early diagnosis of TTP is a key imperative to reduce morbidity and mortality. This article reviews the neurologic complications of TTP and provides neurologists with strategies for diagnosis and treatment.

### 1.1. TTP complicated with cerebrovascular disease

The pathophysiological basis of TTP is the deficiency of ADAMTS13 protease which cleaves the Von Willebrand factor (VWF) polymer. VWF is a protein platelet complex, which forms platelet plug at the site of endovascular injury.^[3]^ Acquired TTP is characterized by development of autoantibodies against ADAMS T3 protease, leading to persistence of VWF polymer and systemic thrombosis. Patients with TTP develop microvascular hemolytic anemia due to the shear force produced by the passage of red blood cells through platelet clusters on the vascular endothelium; this leads to the production of fragmented red blood cells (referred to as mitotic cells).^[4]^

Identification of the first acute TTP episode is challenging owing to the lack of specific clinical symptoms or diagnostic criteria. The diagnosis of TTP mainly depends on various clinical symptoms and laboratory results. Brain is the most common site of ischemic injury in these patients. Most patients with TTP exhibit abnormal neurological manifestations. Patients typically have headache, altered sensorium, mental disorders, ataxia, epilepsy, or focal neurological deficit. Other common symptoms include fever, fatigue, joint pain, myalgia, jaundice, nausea, vomiting, diarrhea, and abdominal pain.

Skin and mucosal bleeding secondary to thrombocytopenia is also common. In the cases reported by Aksay et al,^[5,6]^ patients exhibited renal abnormalities, including oliguria, acute renal failure, proteinuria, and microscopic hematuria.^[5]^ Most patients, develop consumptive thrombocytopenia, and the platelet count is usually less than 20 × 10^9^/L. Extensive deposition of fibrin in blood vessels causes mechanical damage to the red blood cells, resulting in the appearance of fragmented red blood cells in blood smears. Hemoglobin level is usually less than 80 g/L (8 g/dL) secondary to hemolytic anemia. Elevated serum levels of lactate dehydrogenase and bilirubin may represent intravascular hemolysis.^[7]^

Studies have documented the incidence of atypical TTP in patients with stroke. Idowu et al^[8]^ reported a patient with acute cerebral infarction complicated with TTP, who manifested aphasia. Aphasia is one of the classical neurological manifestations in patients with TTP. It is caused by damage to language related areas, leading to severe impairment of language expression despite normal intelligence. After 7 times of therapeutic plasma exchange, the patient’s neurological condition showed significant improvement. Crum et al also reported 2 cases of TTP.^[9]^ The initial findings mainly included focal neurological deficit, including expressive aphasia, which led to delayed diagnosis. Aphasia is commonly caused by brain injury or stroke affecting one or more areas of the brain that process language. Most cases with TTP develop neurological symptoms^[10]^ due to the interaction between platelets and vascular endothelium, which leads to severe coagulation dysfunction. Azmi and Maizuliana reported a 38-year-old woman who presented with TTP, aphasia, and weakness of the right side; this case report also indicated that these symptoms may occur simultaneously.^[11]^ TTP is a blood disorder characterized by neurological and renal abnormalities, fever, anemia, and thrombocytopenia. Most patients with TTP show neurological deficit.^[12]^ CT scan of these patients may show infarction in specific parts of the brain^[13]^

### 1.2. TTP with spinal cord injury

TTP with spinal cord injury is rarely reported. Okhovat et al reported^[14]^ a case of neuromyelitis optica spectrum disease (NMOSD) with TTP in 2017. The patient had hemolytic uremic syndrome; however, since ADAMTS13 of this patient was not measured, it was impossible to determine whether TTP was primary or acquired. These results indicate that both NMOSD and TTP may occur simultaneously; however, the underlying pathogenetic mechanisms are not clear. Here we report a case of myelitis with TTP.

A 57-year-old right-handed woman was first admitted in Nov 2020 with a history of numbness of limbs and sensory changes progressing from both lower extremities to both upper extremities. She had zonesthesia in waist and belly during the previous 1 month; her condition worsened with development of fever and oliguria since 1 day. Therefore, she visited the neurology department of our hospital. Her past medical history was unremarkable for any neurological, rheumatologic and hematological diseases. She had no clinical evidence of sarcoidosis, vasculitis, SLE or Sjo¨gren’s syndrome.

**Figure 1.**
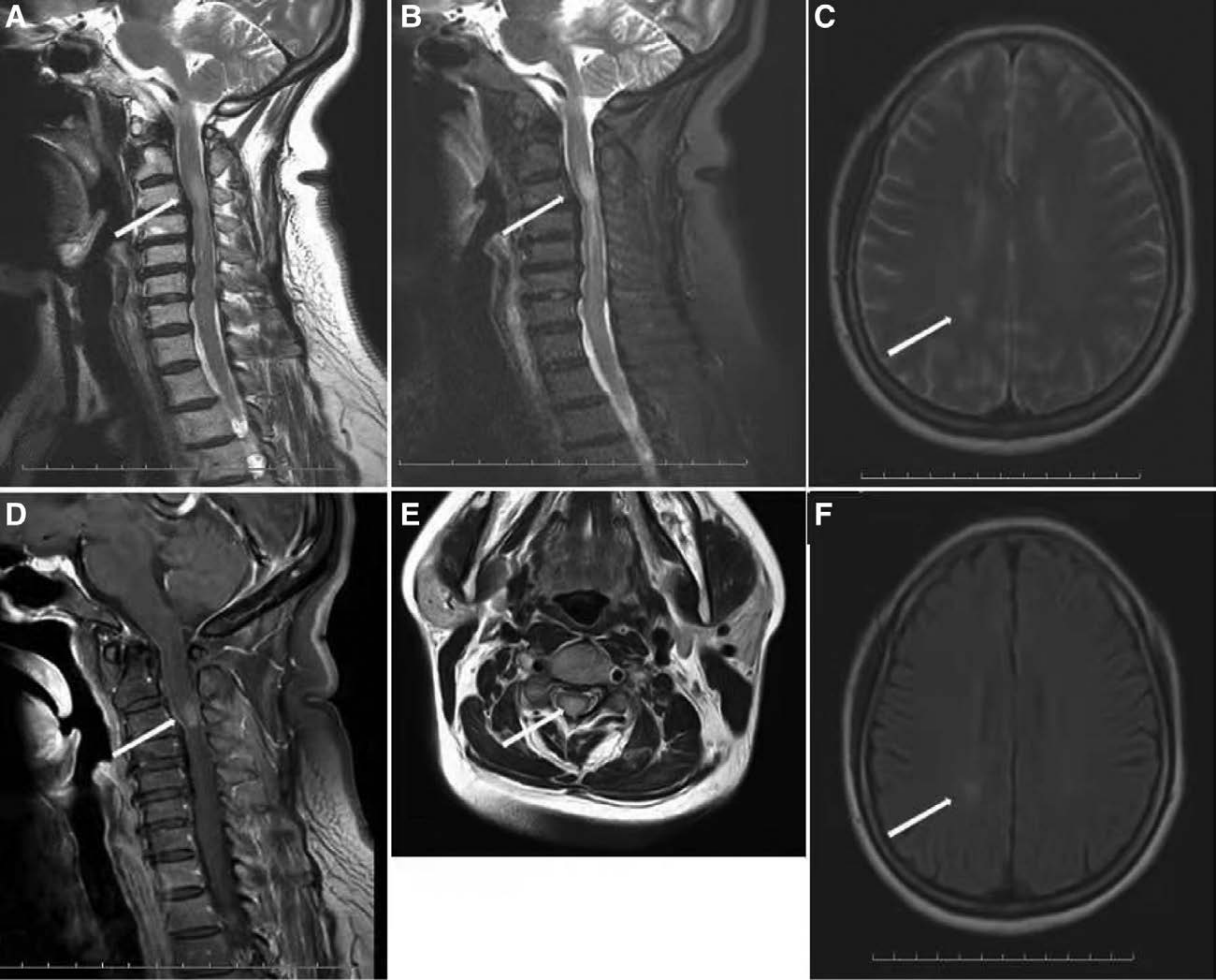
Sagittal magnetic resonance T2-weighted (A) and fat suppression (B) images showing a strip of hyperintensity in the cervical cord (C3–C7) with spinal cord swelling, the edge of the lesion is blurred. Enhanced scan (D) image showing enhancement. Axial T2-weighted (E) image showing small hyperintensities in the cervical cord. Brain axial T2-weighted (C) and Flair (F) images showing 1 nonspecific hyperintense lesion next to the lower corner of lateral ventricles.

On physical examination, she had fever (temperature: 38.6˚C), anemia, and her general condition was poor. Neurologic examination revealed spasticity of upper and lower extremities, decreased muscle power, hyperesthesia in both lower extremities, and hyperreflexia in both upper and lower extremities. Magnetic resonance imaging (MRI) revealed a strip of lesion in the cervical cord (C3–C5); Brain MRI was normal except for 1 nonspecific hyperintense lesion next to the lower corner of lateral ventricles (Fig. 1).

**Figure 2.**
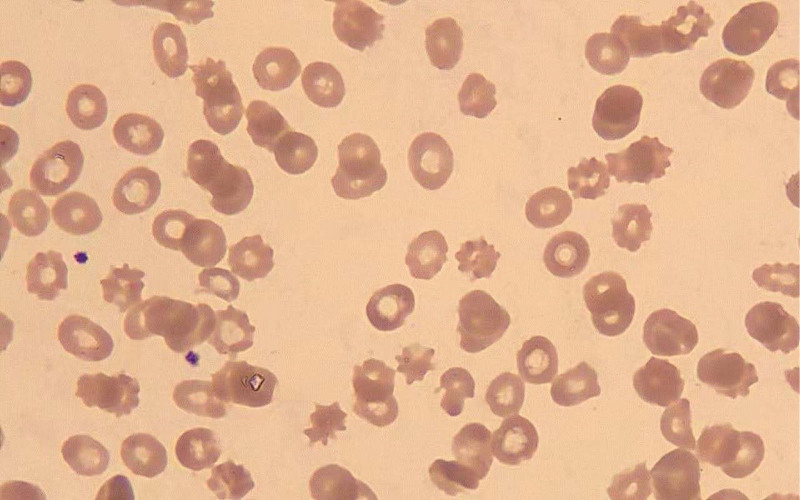
Blood smear showing fragmented erythrocytes and scattered acanthocytes suggesting intravascular hemolysis.

Results of the laboratory tests were unexpected: On cerebrospinal fluid examination, antinuclear antibodies, rheumatoid factor, total complement, and serum angiotensin convertase were normal. AQP4 was positive and MOG was negative. Other laboratory findings were: white blood cell count 9.9 × 10^9^/L (normal: 3.5–9.5 × 10^9^/L); hemoglobin, 92 g/L (normal: 130–175 g/L); hematocrit 32% (40%–50%); platelet count 20 × 10^9^/L (normal: 125–350 × 10^9^/L); mean corpuscular volume: 88; reticulocytes: 22.0% (normal 0.5%–1.5%); serum creatinine: 500 µmol/L (55–111 µmol/L), blood urea nitrogen: 38.6 mmol/L (normal: 3.6–9.5); urine protein (+ +); urine red blood cells + 3; serum direct bilirubin: 31.1 µmol/L (normal: 0.0–6.84 µmol/L); prothrombin time (PT): 16.3 seconds (normal: 11.0–15.0 seconds); activated partial thromboplastin time: 55.1 sec (normal: 28.0–42.5 seconds), fibrinogen: 809 mg/dL (normal: 200–400 mg/dL); international normalized ratio (INR): 1.31; D-dimer: 3.07 µg/mL (normal: 0–0.5); troponin: 0.131 ng/mL. The patient had increased lactate dehydrogenase, thrombocytopenia, renal insufficiency, and hemolytic anemia. Blood smear examination showed fragmented red blood cells and scattered acanthocytes (Fig. 2), which were common abnormal form in hemolytic anemia. The clinical diagnosis was NMOSD, idiopathic thrombocytopenic purpura (TTP), renal insufficiency, hemolytic anemia. According to the literature, we calculated the probability of severe deficiency of ADAMTS13^[15]^; her PLASMIC score was 6 (Table 1), indicating a high risk of severe deficiency of ADAMTS13. The patient was in critical condition and was treated in the intensive care unit. The patient did not respond to hormone shock (1000 mg/D), platelet transfusion, and anti-infection treatment, and died 3 days after admission.

**Table 1:** Components of PLASMIC score^[15]^ and patient data.

Component	Our patient	Point
Platelet count <30 × 10^9^/L	20 × 10^9^/L	1
Hemolysis (indirect bilirubin > 2 mg/dL, uncorrected reticulocyte count > 2.5%, or undetectable haptoglobin)	Indirect bilirubin 3.11 mg/dL, uncorrected reticulocyte 22.0%	1
No active cancer in the previous yr	No active cancer in the previous yr	1
No history of solid organ or stem cell transplant	No history of solid organ or stem cell transplant	1
Mean corpuscular volume < 90 fL	Mean corpuscular volume 88 fL	1
International normalized ratio < 1.5	International normalized ratio 1.31	1
Creatinine < 2 mg/dL	Creatinine 5 mg/dL	0
Total score: 6		

The concurrent occurrence of NMOSD and TTP in this patient is consistent with the characteristics of acquired autoimmunity. In previously reported cases with central nervous system damage, renal injury was more common and prominent^[16]^ while spinal cord injury was rarely reported. Previous reports have described the concurrent occurrence of NMOSD and other autoimmune diseases (such as systemic lupus erythematosus and Sjogren’s syndrome).^[17]^ This patient likely had acquired and immune-mediated TTP. However, it is possible that the patient had concomitant TTP and NMOSD as 2 different autoimmune diseases. This case highlights the potential concomitant occurrence of NMOSD and TTP; early diagnosis can help prevent morbidity and mortality in these patients.

### 1.3. TTP complicated with other central nervous system damage

In a study by Benjamin et al,^[18]^ the prevalence of stroke in TTP patients was 13.9%, which was higher than that in the general elderly population. The reported prevalence in the US population in the age-group of 60 to 79 years was 6.3%,^[18]^ while that in a large Italian cohort of older people was 7.8%.^[19]^ Patients with TTP were found to have a high prevalence of obesity at the time of initial diagnosis.^[20]^ An increasing body of evidence^[20–24]^ suggests that TTP patients may have a higher risk of anxious-depressive symptoms and cognitive decline. This phenomenon may be attributable to episodes of neurological complications, risk of relapse, the complex assistance needs, and the related psychosocial implications.

Szamosi et al^[25]^ reported a case of TTP complicated with PRES, who also had SLE. According to the literature, this case is similar to the previously reported case in terms of neurological symptoms, and it is also the first case of PRES syndrome and TTP reported by Hungarian scholars. The diagnosis was based on clinical symptoms and neuroimaging findings. The brain edema in PRES is usually reversible, and the imaging signs of improvement typically lag the recovery of clinical symptoms. MRI is helpful in identifying patients with poor prognosis. Diffusion weighted imaging can reliably differentiate between angiogenic edema and cytotoxic edema during cerebral ischemia. If the diffusion weighted imaging signal is too strong, it is suggestive of cytotoxic edema, and death may occur due to progressive edema or cerebral hemorrhage.^[26]^ Suspected cytotoxic edema and hemorrhage may be associated with TTP. Continuation of conventional dose of immunosuppressive therapy is recommended along with initiation of antihypertensive therapy. High-dose corticosteroids may predispose patients to high blood pressure and fluid overload and are not recommended.^[27]^

## 2. Summary

Atypical clinical presentation of TTP poses a diagnostic challenge and prevents prompt treatment. Therefore, we highlight the importance of early recognition of TTP in patients with atypical presentation who may not have the expected clinical or laboratory findings. This is particularly important in TTP patients with other concomitant autoimmune diseases or age-related comorbid conditions such as osteoporosis, arterial hypertension, ischemic heart disease, and cerebrovascular disease.

## Acknowledgments

We thank the patient and the donor for provision of clinical data.

## Author contributions

HZ collected and analyzed the data, and wrote the manuscript. JZ and JYL supervised the analyses and manuscript preparation. All authors discussed and interpreted the results. All authors read and approved the final manuscript.

**Project administration:** Jing-Yao Liu.

**Writing – original draft:** Hui Zhu.

**Writing – review & editing:** Hui Zhu.
